# Increasing the Spatial Resolution of 3T Carotid MRI Has No Beneficial Effect for Plaque Component Measurement Reproducibility

**DOI:** 10.1371/journal.pone.0130878

**Published:** 2015-07-10

**Authors:** Diederik F. van Wijk, Aart C. Strang, Raphael Duivenvoorden, Dirk-Jan F. Enklaar, Aeilko H. Zwinderman, Rob J. van der Geest, John J. P. Kastelein, Eric de Groot, Erik S. G. Stroes, Aart J. Nederveen

**Affiliations:** 1 Department of Vascular Medicine, Academic Medical Center, Amsterdam, the Netherlands; 2 Department of Clinical Epidemiology and Biostatistics, Academic Medical Center, Amsterdam, the Netherlands; 3 Department of Radiology, Leiden University Medical Center, Leiden, the Netherlands; 4 Department of Radiology, Academic Medical Center, Amsterdam, the Netherlands; University Hospital Würzburg, GERMANY

## Abstract

**Purpose:**

Different in-plane resolutions have been used for carotid 3T MRI. We compared the reproducibility, as well as the within- and between reader variability of high and routinely used spatial resolution in scans of patients with atherosclerotic carotid artery disease. Since no consensus exists about the optimal segmentation method, we analysed all imaging data using two different segmentation methods.

**Materials and Methods:**

In 31 patient with carotid atherosclerosis a high (0.25 × 0.25 mm^2^; HR) and routinely used (0.50 × 0.50 mm^2^; LR) spatial resolution carotid MRI scan were performed within one month. A fully blinded closed and a simultaneously open segmentation were used to quantify the lipid rich necrotic core (LRNC), calcified and loose matrix (LM) plaque area and the fibrous cap (FC) thickness.

**Results:**

No significant differences were observed between scan-rescan reproducibility for HR versus LR measurements, nor did we find any significant difference between the within-reader and between-reader reproducibility. The same applies for differences between the open and closed reads. All intraclass correlation coefficients between scans and rescans for the LRNC, calcified and LM plaque area, as well as the FC thickness measurements with the open segmentation method were excellent (all above 0.75).

**Conclusions:**

Increasing the spatial resolution at the expense of the contrast-to-noise ratio does not improve carotid plaque component scan-rescan reproducibility in patients with atherosclerotic carotid disease, nor does using a different segmentation method.

## Introduction

Primary and secondary prevention of cardiovascular disease is a major public health priority. The long-term, slowly progressive character of atherosclerosis offers an opportunity for early detection and treatment. Although statin therapy has proved highly effective in reducing the risk of cardiovascular events[1], residual risk remains high, and early accurate risk assessment combined with the ongoing development of novel pharmacological agents is needed to further improve primary and secondary prevention strategies.

Improvement of MRI carotid artery measurements is thought to contribute to earlier detection of disease, improvement of risk stratification algorithms and more reliable, individual monitoring of disease progression. In addition, clinical trials using MRI carotid artery measurements as a surrogate endpoint[2–4] can assess the efficacy of newly developed pharmacotherapies in a relative early stage of the developmental process, guiding further possible large multi-center endpoint trials. High-resolution MRI enables accurate and highly reproducible measurement of increased carotid artery wall dimension measurements.[5,6] However, the occurrence of stroke in patients with mild to moderate (<70%) carotid stenosis suggests that lumen narrowing is not the strongest classifier of atherosclerotic disease severity.[7] In line, high-risk features of atherosclerosis, such as intraplaque hemorrhage and a lipid rich necrotic core[4] (LRNC), have been reported across all stenotic categories (0%–99%).[8,9] In fact, complex lesions develop in substantial numbers in the absence of high-grade stenosis.[8]

In addition to carotid artery wall dimensions measurements, multi-contrast MRI protocols can accurately determine plaque components[5,10,11], although no consensus exists regarding the most accurate segmentation method and reading methodology and so far plaque component segmentation has proven to be too difficult to become part of the standard workup. Saam et al. previously determined the scan-rescan reproducibility of carotid plaque components measurements at 1.5 T in a severely diseased population using an open-simultaneously manual segmentation method between the repeated measurements.[12] More recently, Li et al. investigated the scan-rescan reproducibility in a broader population at 3 Tesla, only analyzing components present in at least one-time point.[13] Both studies were performed with an in-plane resolution of approximately 0.50 × 0.50 mm.[6] These resolution parameters are commonly used in carotid MRI as a tradeoff between resolution, signal-to-noise ratio (SNR), and acquisition time. These three imaging parameters are highly interdependent: higher resolution allows observation of smaller details, but typically reduces SNR, and increases imaging time. While maintaining the same resolution, it was shown that 3T MRI yields superior SNR compared to 1.5T MRI scanners[14–16]. In addition, Balu et al. demonstrated that an 8 instead of a 4 channel coil configuration led to an additional improvement of the SNR for carotid plaque scanning.[17]

In an effort to further improve MRI carotid plaque component measurements, we hypothesized that increasing the in-plane spatial resolution using a 3T MRI scanner equipped with a dedicated 8 channel carotid coil would improve carotid plaque component scan-rescan reproducibility, despite the expected lower SNR. Secondly, since no consensus exists for the optimal segmentation method we compared two different manual segmentation methods for carotid plaque component image analysis. For this aim, we performed prospective repeated 3T MRI scans with a dedicated 8 channel carotid coil at 0.50 × 0.50 mm^2^ and 0.25 × 0.25 mm^2^ in-plane resolution in patients with overt carotid atherosclerosis.

## Materials and Methods

### Ethics statement

The review board of the Academic Medical Center in Amsterdam, the Netherlands approved the study protocol. Written informed consent was obtained from all participants. This study has been conducted according to the principles expressed in the Declaration of Helsinki.

### Participants

Individuals with one or more atherosclerotic events were screened for the presence of significant atherosclerotic disease in one of the carotid arteries using echo duplex measurements. Individuals with 30 to 70% stenosis of the carotid artery were included. All participants were scheduled for a scan and a rescan within one month. Scans were performed between September 2009 and April 2011.

### 3T MRI

MRI scans were obtained on a 3T whole-body scanner (Intera, Philips Medical Systems, Best, The Netherlands), with the use of an 8 channel dedicated bilateral carotid artery coil (Shanghai Chenguang Medical Technologies, Shanghai, China). Positioning of the image stack was performed using axial magnetic resonance angiography images acquired with a time of flight (TOF) sequence covering the carotid arteries at both sides (field of view (FOV) 10 × 10 cm, 40 slices of 2 mm thickness). These images together with the ultrasound duplex data were used for planningtheT1w, T2w, PDw and TOF sequences using a FOV of 6 × 6 cm at the center of the carotid plaque in the carotid artery with the most profound plaque burden. This could be either side of the neck. Subsequently, 8 slices of T1w, T2w, PDw and TOF images of 2 mm thickness were acquired using ECG-gated unilateral axial turbo spin echo sequences for both the HR and LR images during each scan session. Overview images showing the image stacks superimposed over the carotid artery were used to to plan the repeat scan. Images were saved according to the DICOM protocol. Standardized equipment and protocols were used for image storage and data management. Before any quantitative analysis was performed one reader (RD) corrected all scan and rescan images for possible Z-axis displacement using T1w, PDw and TOF images. After localisation of the carotid bifurcation both proximal and caudal scan and rescan images were compared. For this study low resolution (LR) and high resolution measurements (HR) were compared. Therefore PDw, T2w, T1w and TOF sequences were repeated for the two different resolutions at each scan session. The non-interpolated pixel size of 0.5 × 0.5 mm^2^ is referred to as the low resolution (LR) and the non-interpolated pixel size of 0.25 × 0.25 mm^2^ is referred to as the high resolution (HR) setting. Table 1 displays the full scan parameters.

**Table 1:** Scan parameters for the HR and LR carotid arterial wall dimension measurements. HR = high resolution; LR = low resolution; TSE = turbo spin-echo, FFE = fast field echo, FOV = field of view, DIR = double inversion-recovery, NEX = number of excitations.

	High Resolution (HR)				Low Resolution (LR)			
Parameters	Black-blood PDw	Black-blood T2w	Black-blood T1w	Bright blood TOF	Black-blood PDw	Black-blood T2w	Black-blood T1w	Bright blood TOF
Sequence	TSE	TSE	TSE	FFE	TSE	TSE	TSE	FFE
ECG gating	end diastole	end diastole	end diastole	gate delay 200 ms	end diastole	end diastole	end diastole	gate delay 200 ms
Image mode	2D	2D	2D	2D	2D	2D	2D	2D
Scan plane	Axial	Axial	Axial	Axial	Axial	Axial	Axial	Axial
TR (ms)	2 heart beats	2 heart beats	1 heart beat	35	2 heart beats	2 heart beats	1 heart beat	19
TE (ms)	8	50	8	7	8	50	8	5
ETL	12	12	8	-	12	12	8	-
FOV (mm)	60 × 60	60 × 60	60 × 60	60 × 60	60 × 60	60 × 60	60 × 60	60 × 60
Matrix size	240 × 240	240 × 240	240 × 240	240 × 240	120 × 120	240 × 240	120 × 120	120 × 120
Resolution (mm)	0.25 × 0.25	0.25 × 0.25	0.25 × 0.25	0.25 × 0.25	0.5 × 0.5	0.5 × 0.5	0.5 × 0.5	0.5 × 0.5
Slice thickness (mm)	2	2	2	2	2	2	2	2
Flip angle	90	90	90	20	90	90	90	20
Number of slices	8	8	8	8	8	8	8	8
Blood suppression	DIR	DIR	DIR	Inflow suppression (veins)	DIR	DIR	DIR	Inflow suppression (veins)
Fat suppression	SPAIR	SPAIR	SPAIR	-	SPAIR	SPAIR	SPAIR	-
NEX	1	1	1	1	1	1	1	1
Scan time (minutes)*	6.6	6.6	4.4	2	3.3	3.3	2.2	1

* Scan times at heart rate of 60 min^-1^

### 3T MRI Image Analysis

A fully blinded segmentation method was compared to a segmentation method with side-by-side workstations enabling a direct comparison between the scan and rescan images. With the fully blinded segmentation method (referred to as closed segmentation), the scans and rescans were analysed one-by-one, in a fully random order, without knowledge of patient characteristics and scan session. With the side-by-side workstation segmentation method (referred to as open segmentation) scan and rescan were analysed simultaneously, without knowledge of patient characteristics and scan session. To prevent recall bias, the open segmentation method was performed two months after the last blinded analysis.

Delineation of LRNC, calcification and loose matrix (LM) boundaries were performed manually with the aid of a dedicated vessel wall analysis package VesselMass (VesselMass, Leiden University Medical Center, Leiden, The Netherlands). [18] All four weightings were used to identify the different plaque components according to previously published literature.[10] Briefly, isointense to hyperintense areas on T1w and PDw images with varying intensities on T2w and TOF images was considered to correspond with the LRNC. Fresh intraplaque hemorrhage appears as a hyperintense signal on T1W and TOF images and as an isointense signal on T2w/PDw images. Recent hemorrhage is identified by a hyperintense signal on all 4 contrast weightings. Calcification was defined by a hypointense signal on all 4 weightings. Loose matrix was delineated when plaques had hyperintense areas on T2w and PDw, isointense to hypointense areas on T1w and isointense areas on TOF images. Fibrous cap was identified if a high signal area adjacent to the lumen on T2w images was present[19,20]. In TOF images, fibrous cap was considered present if a hyposignal band adjacent to the lumen on the plaque surface was present[21]. In the absence of a LRNC, fibrous cap cannot be distinguished from the fibrous component on T2w images. Therefore, images where no LRNC was identified in the scan and rescan images were excluded from this analysis. The thickness of the fibrous cap was measured in the area directly overlying the thickest part of the underlying lipid rich necrotic core[22]. If scan and rescan images during the open segmentation analysis could not be interpreted similarly according to these criteria, they were discussed in an expert meeting for a definite decision. If no agreement could be reached in accordance with the image delineation strategy outlined above the scan and rescan images were analysed unchanged independent of a possible mismatch between scan and rescan images.

To assess the between-reader reproducibility of the closed segmentation method, two readers independently analysed all (both HR and LR scans and rescans) fully blinded MRI image stacks. To assess the within-reader reproducibility, one reader blinded to previous outcome data, analysed all (both HR and LR scans and rescans) fully blinded image stacks in a second session, 2 months after the first analysis.

Signal-to-noise ratios (SNR) were calculated as an internal control on T1w images and defined as SNR = *S/σ*, where *S* is the true signal intensity corrected for the noise contribution and σ is the true SD of the noise. σ was calculated from the measured SD of the noise (SD_n_) and the numbers of receivers: SD_n_ = 0.7σ. Corrected signal intensity *S* was obtained from the measured magnitude signal (*S*_m_) and the measured magnitude of the background noise (*S*_n_): *S* = (*S*_m_^2^-*S*_n_^2^)^1/2^. The magnitude (*S*_n_) and the SD (SD_n_) of the background noise were measured in the corner of the image in a region free of signal and free of artefacts. Contrast-to-noise ratios (CNR) between wall and lumen were calculated as CNR = SNR_wall_-SNR_lumen_.

### Statistical Analysis

Continuous variables are expressed as mean ± SD. The SD of the paired differences (SDpd) between the initial and the rescan were calculated for the LRNC, calcification and LM plaque area. A paired t-test was used to test for differences in plaque component surface areas between the HR and the LR scan and rescan(i.e. HR scan versus LR scan; HR rescan versus LR rescan). Bland-Altman plots were used to illustrate systematic bias between scan and rescan images.[23] The agreement between successive MRI scans, as well as the between observer and within observer agreement was assessed using intra-class correlation coefficients (ICC). An ICC of <0.40 indicated poor, one between 0.40 and 0.75 indicated fair to good, and one of >0.75 indicated excellent scan-rescan reproducibility.[24] To investigate differences between the HR and LR reproducibility of the plaque components we calculated the ICCs for the LRNC, calcified plaque and LM plaque area, as well as the FC thickness measurements between the HR and LR scan-rescan and between the open and closed segmentation method. In addition a Levene’s test was used to test for statistical significant differences of the HR and LR scan-rescan variances for the different plaque components. All statistical analyses were performed using PASW statistics 18.0 for Windows (SPSS Inc., Chicago, IL, USA).

## Results

Fifty-one individuals with one or more atherosclerotic events were screened for the presence of significant atherosclerotic disease in one of the carotid arteries. Thirty-one individuals with a 30 to 70% stenosis of the carotid artery were included. Full baseline characteristics of the patient population are listed in Table 2. All participants were scheduled for a scan and a rescan session. Two patients cancelled the second appointment and withdrew consent. One LR scan could not be completed due to a panic attack, whereas two other subjects did not complete their LR scan due to discomfort. The average interval between the scan and rescan sessions was 26 days. No significant clinical events were reported during the study. Prior to the analyses, 2 HR and 1 LR data sets were excluded based on a poor image quality. After exclusion, 27 full repetitive sets of the HR scans, and 25 repetitive sets of the LR scans were available for the final analyses. This corresponds to 832 initial available images of which 808 images (97%) were of sufficient quality to be analysed. From these images twenty-one image stacks of the available 101 needed a Z-axis correction. Fourteen image stacks were shifted by one slice, 3 image stacks by two slices, 3 image stacks by three slices and 1 image stack needed a Z-axis correction of 4 slices. Table 3 displays the number of components identified on the scan and rescan images according to the resolution and stratified by segmentation method.

**Table 2:** Baseline characteristics of the study population. Data are presented as mean ± (SD) or numbers with the corresponding percentage. Triglyceride and CRP concentrations are presented as median with the [25^th^ to 75^th^ percentile]. LDL = low-density lipoprotein; HDL = high-density lipoprotein; HbA1c = glycosylated hemoglobin; CRP = C-reactive protein. ACE = Angiotensin converter enzyme; AT II = Angiotensin receptor; IMT_CC_ = common carotid intima-media thickness; IMT_BULB_ = carotid bulb intima-media thickness; IMT_ICA_ = internal carotid artery intima-media thickness.

**Patient characteristics**	
Patients, n	N = 31
Age, years	68.8 ± 7.5
Women, n (%)	15 (48)
Body mass index, kg/m2	25.4 ± 2.9
Current smoker, n (%)	7 (23)
Diabetes mellitus, n (%)	7 (23)
Hypertension, n (%)	15 (48)
Myocardial infarction, n (%)	7 (23)
Stroke, n (%)	14 (45)
Peripheral artery disease, n (%)	6 (19)
Systolic blood pressure, mmHg	144 ± 20
Diastolic blood pressure, mmHg	73 ± 11
Total cholesterol, mmol/l	4.86 ± 1.10
LDL-cholesterol, mmol/l	2.85 ± 1.16
HDL-cholesterol, mmol/l	1.45 ± 0.45
Triglycerides, mmol/l	1.20 [0.88–1.63]
HbA1c, %	6.28 ± 0.96
CRP, mg/L	1.6 [1.0–3.8]
**Medication use**	
ACE-inhibitors, n (%)	4 (13)
AT II-inhibitors, n (%)	5 (16)
Anticoagulants, n (%)	26 (84)
Biguanides, n (%)	3 (10)
ß-blockers, n (%)	10 (32)
Calcium channel blockers, n (%)	7 (23)
Cardiac glycosides, n (%)	1 (3)
Diuretics, n (%)	4 (13)
Insuline, n (%)	3 (10)
Statins, n (%)	23 (74)
**Ultrasound dimension measurements measurementsaracteristics**	
IMT_CC_, mm	1.10 (0.56)
IMT_BULB_, mm	1.73 (0.72)
IMT_ICA_, mm	1.02 (0.56)
**MRI dimension measurements**	
Total wall volume, mm^3^	735.0 (431.8)
Mean wall area, mm^2^	53.0 (29.6)
Normalized wall index	0.659 (0.099)
Mean wall thickness, mm	2.07 (0.63)

**Table 3:** Number of identified carotid plaque components with corresponding scan-rescan mismatch. Data are presented as number.

	High Resolution		Low Resolution	
	Scan-rescan	Mismatch	Scan-rescan	Mismatch
**Total scans available**	27	-	25	-
**LRNC**				
Closed segmentation	10	5	15	7
Open segmentation	9	2	9	2
**Calcified plaque**				
Closed segmentation	25	6	24	4
Open segmentation	21	1	21	1
**Loose matrix**				
Closed segmentation	18	11	19	6
Open segmentation	14	2	16	0

### HR scanning decreases the SNR of carotid MRI dimension measurements

As expected we measured a significantly lower mean SNR of the arterial wall for the HR compared to the LR carotid measurements (17.8 ± 8.1 vs. 52.2 ± 13.4; p <0.001). We found a mean contrast-to-noise ratio (CNR) between the arterial wall and arterial lumen of 15.7 ± 7.2 for the HR and of 48.0 ± 13.1 for the LR carotid measurements (p <0.001).

### Plaque component measurements

**Fig 1:**
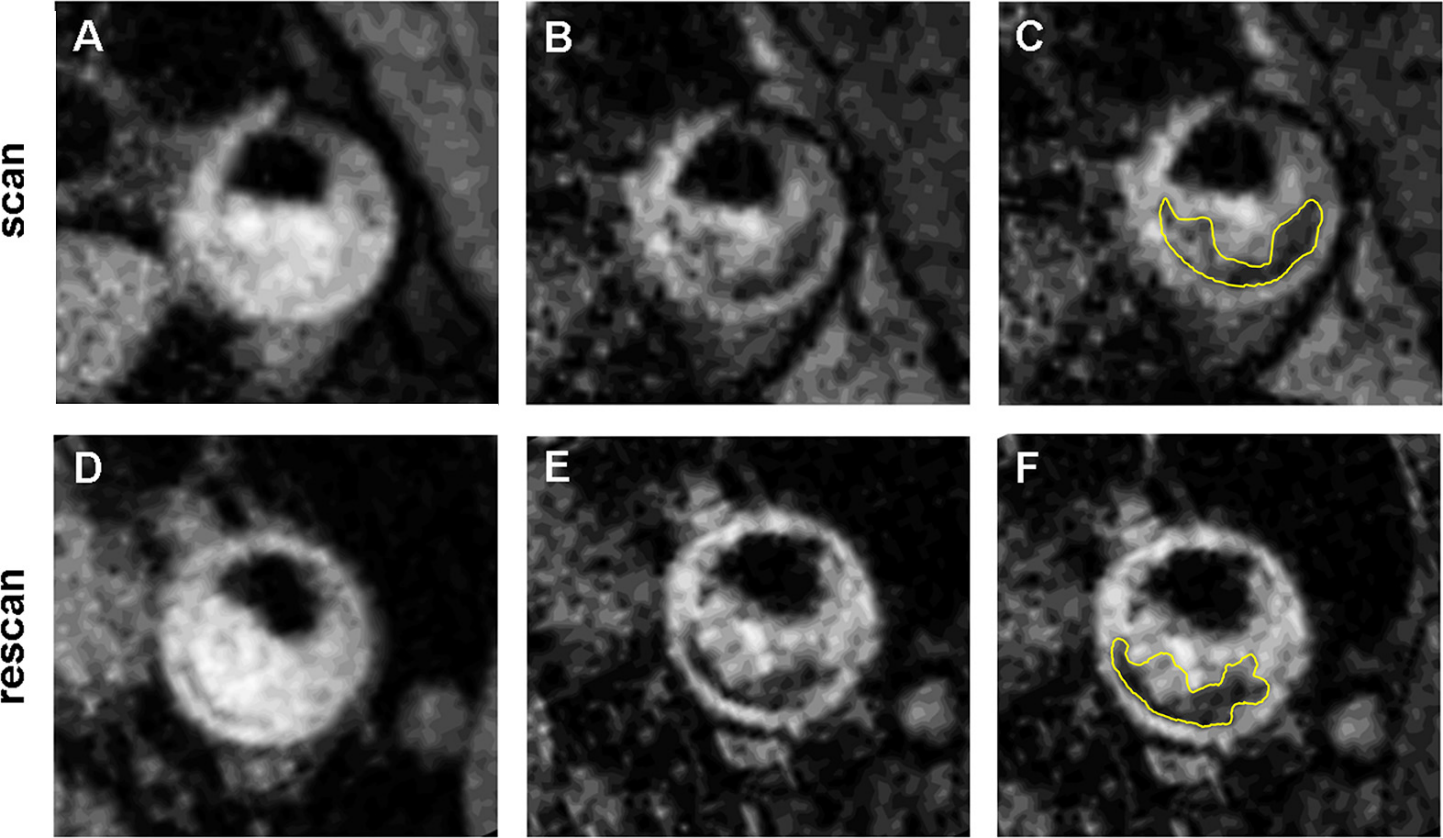
Representative sample of scan and rescan HR images containing a LRNC. Panel A and B show the difference in intensity between a T1w (panel A, panel D) and a T2w (panel B, panel E) image for the LRNC of a scan and rescan respectively. Panel C (scan) and panel F (rescan) show the manual delineation of the LRNC with the closed segmentation method.

**Fig 2:**
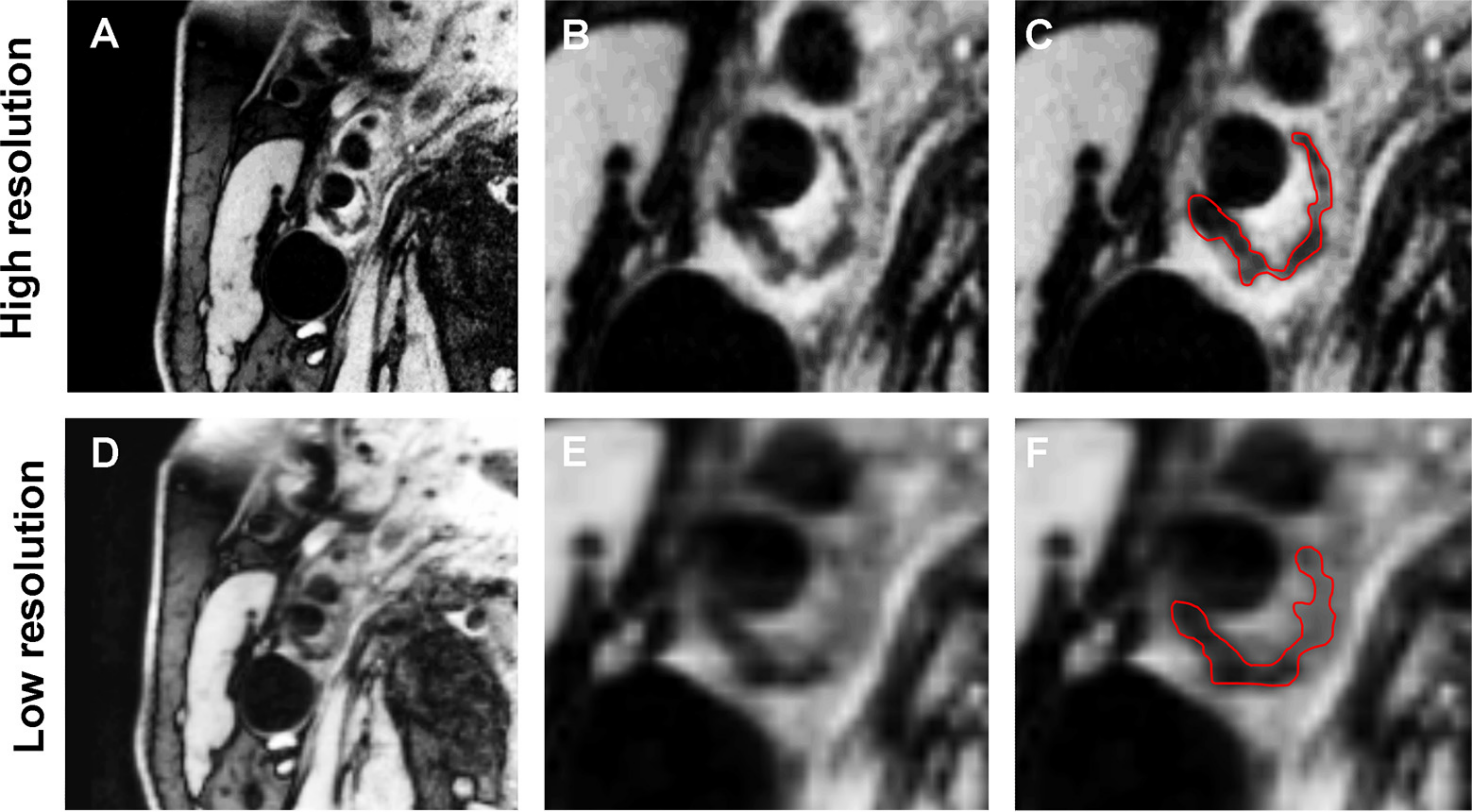
Representative sample of a HR and LR scan containing a calcified plaque area. Panel A and B show a HR image at a different magnification, with a manual delineated calcium contour obtained with the closed segmentation method (panel C). Panel D and E show a LR image at a different magnification, with a manual delineated calcium contour obtained with the closed segmentation method (panel F).

Tables 4, 5, 6 and 7 comprises the LRNC plaque area, the FC thickness and the calcified and loose matrix plaque area for the HR and LR scans. We found no differences between the LRNC area, calcified plaque area and FC thickness between the HR and LR scans. Plaque loose matrix areas are significant smaller for the HR measurements compared to the LR measurements for the open (p<0.05) and closed (p<0.05) segmentation method. Fig 1 shows an example of a HR scan and rescan containing a LRNC, whereas Fig 2 shows a calcified plaque area on a HR and LR scan. We only found one intraplaque hemorrhage which was not identified in the repeat scan and therefore excluded from the statistical analysis.

**Table 4:** Open and closed segmented LRNC surface area measurements for HR and LR carotid arterial wall measurements. Data are presented as mean with ± SD. ICCs are given with the corresponding 95% confidence interval.

	HR	LR	p-value
**Scan measurements**			
Scan measurements (mm^2^)			
Closed segmentation	6.2 (5.8)	4.6 (5.2)	
Open segmentation	5.7 (4.4)	6.2 (5.7)	0.43*
Rescan measurements (mm^2^)			0.91†
Closed segmentation	5.6 (5.2)	5.7 (4.9)	
Open segmentation	6.8 (4.5)	5.1 (5.5)	
**Variablity**			
Paired differences (mm^2^)			
Closed segmentation	0.6 (6.2)	1.1 (4.1)	
Open segmentation	1.1 (2.7)	1.1 (1.4)	
**Reproducibility**			
ICC			
Closed segmentation	0.58 (0.0–0.81)	0.80 (0.43–0.93)	0.50‡
Open segmentation	0.89 (0.57–0.98)	0.98 (0.90–0.99)	0.67‡

* = p-value for paired t-test between HR scan and LR scan.

† = p-value for paired t-test between HR rescan and LR rescan using the closed segmentation method.

‡ = p-value for Levene’s test between HR and LR measurements for the corresponding segmentation. HR = high resolution; LR = low resolution; ICC = intraclass correlation coefficient; SD = standard deviation.

**Table 5:** Open and closed fibrous cap thickness measurements for HR and LR carotid arterial wall measurements. Data are presented as mean with ± SD. ICCs are given with the corresponding 95% confidence interval.

	HR	LR	p-value
**Scan measurements**			
Scan measurements (mm)			
Closed segmentation	1.3 (0.7)	0.9 (0.6)	
Open segmentation	1.3 (0.5)	1.2 (0.6)	0.42*
Rescan measurements (mm)			0.70†
Closed segmentation	1.0 (0.7)	1.2 (0.9)	
Open segmentation	1.3 (0.5)	1.1 (0.5)	
**Variablity**			
Paired differences (mm)			
Closed segmentation	0.3 (0.4)	0.3 (0.6)	
Open segmentation	0.0 (0.2)	0.0 (0.1)	
**Reproducibility**			
ICC			
Closed segmentation	0.91 (0.33–0.99)	0.84 (0.28–0.97)	0.54‡
Open segmentation	0.96 (0.81–0.99)	0.98 (0.91–0.99)	0.09‡

* = p-value for paired t-test between HR scan and LR scan.

† = p-value for paired t-test between HR rescan and LR rescan using the closed segmentation method.

‡ = p-value for Levene’s test between HR and LR measurements for the corresponding segmentation. HR = high resolution; LR = low resolution; ICC = intraclass correlation coefficient; SD = standard deviation.

**Table 6:** Open and closed segmented calcified surface area measurements for HR and LR carotid arterial wall measurements. Data are presented as number with percentage or mean with SD. ICCs are given with the corresponding 95% confidence interval.

	HR	LR	p-value
**Scan measurements**			
Scan measurements (mm^2^)			
Closed segmentation	3.8 (5.5)	5.0 (7.7)	
Open segmentation	4.2 (6.0)	4.7 (5.9)	0.59*
Rescan measurements (mm^2^)			0.58†
Closed segmentation	3.7 (5.0)	4.5 (6.5)	
Open segmentation	4.4 (6.0)	4.8 (6.0)	
**Variablity**			
Paired differences (mm^2^)			
Closed segmentation	0.1 (2.7)	0.5 (3.5)	
Open segmentation	0.2 (1.3)	0.1 (0.9)	
**Reproducibility**			
ICC			
Closed segmentation	0.93 (0.85–0.97)	0.94 (0.86–0.97)	0.41‡
Open segmentation	0.99 (0.98–0.99)	0.99 (0.99–1.00)	0.73‡

* = p-value for paired t-test between HR scan and LR scan.

† = p-value for paired t-test between HR rescan and LR rescan using the closed segmentation method.

‡ = p-value for Levene’s test between HR and LR measurements for the corresponding segmentation. HR = high resolution; LR = low resolution; ICC = intraclass correlation coefficient; CV = coefficient of variation; SD = standard deviation.

**Table 7:** Open and closed segmented loose matrix surface area measurements for HR and LR carotid arterial wall measurements. Data are presented as mean with ± SD. ICCs are given with the corresponding 95% confidence interval.

	HR	LR	p-value
**Scan measurements**			
Scan measurements (mm^2^)			
Closed segmentation	4.6 (4.8)	8.5 (6.7)	
Open segmentation	5.7 (4.8)	9.5 (5.9)	0.02*
Rescan measurements (mm^2^)			0.05†
Closed segmentation	4.3 (5.2)	7.2 (7.1)	
Open segmentation	6.9 (4.8)	8.9 (5.9)	
**Variablity**			
Paired differences (mm^2^)			
Closed segmentation	0.4 (4.5)	1.3 (6.1)	
Open segmentation	1.1 (1.6)	0.7 (2.4)	
**Reproducibility**			
ICC			
Closed segmentation	0.76 (0.40–0.91)	0.76 (0.40–0.91)	0.07‡
Open segmentation	0.97 (0.91–0.99)	0.96 (0.88–0.99)	0.09‡

* = p-value for paired t-test between HR scan and LR scan.

† = p-value for paired t-test between HR rescan and LR rescan using the closed segmentation method.

‡ = p-value for Levene’s test between HR and LR measurements for the corresponding segmentation. HR = high resolution; LR = low resolution; ICC = intraclass correlation coefficient; CV = coefficient of variation; SD = standard deviation.

### Scan-rescan reproducibility and variability of plaque component measurements

**Fig 3:**
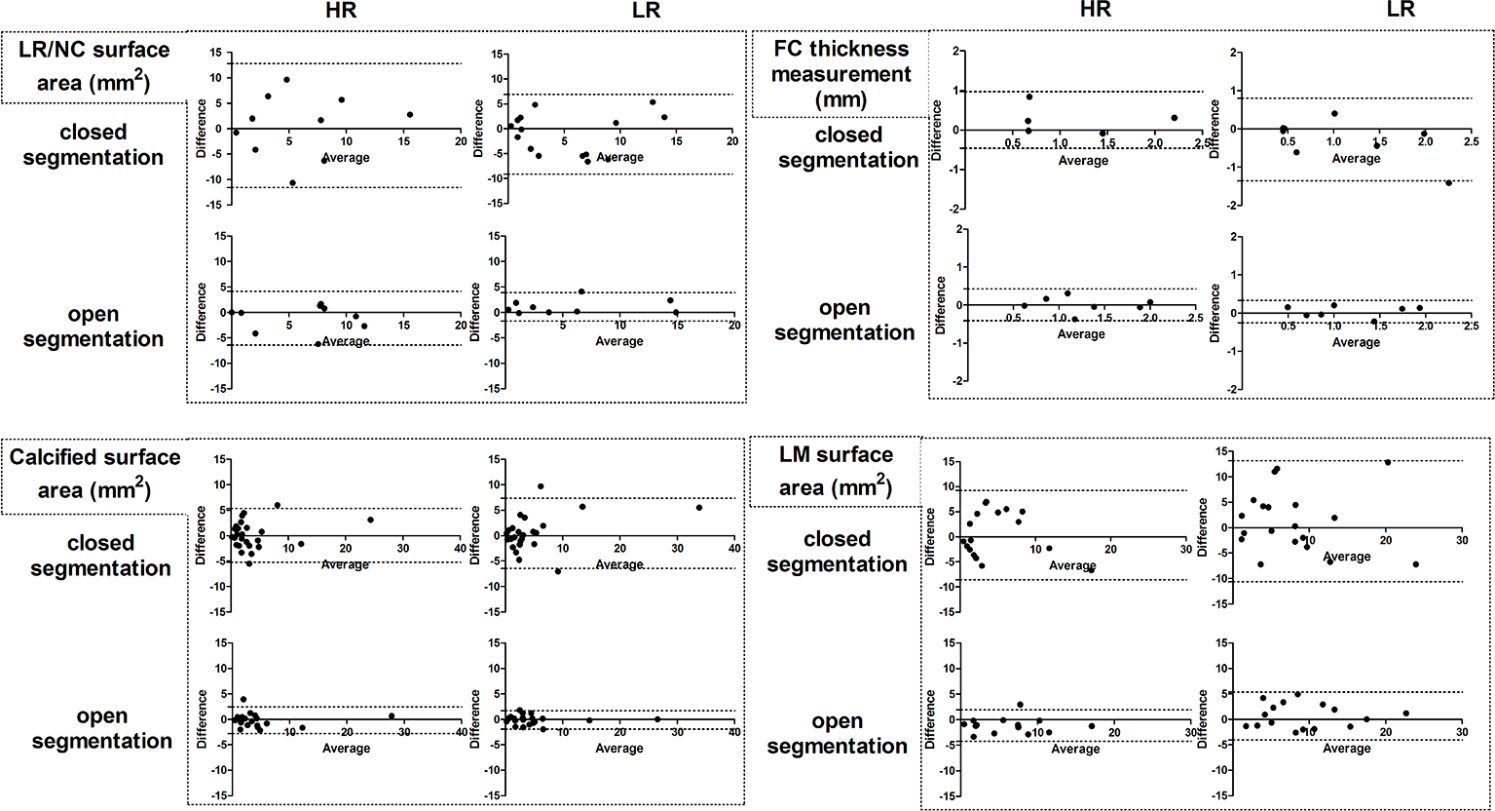
Bland-Altman plots of the high resolution and low resolution carotid plaque composition measurements for all parameters and for the open and closed segmentation method. The middle dashed line of each plot indicate the bias. The upper and lower dashed line indicate the 95% limits of agreement.

Using a paired t-test no significant differences were found between the HR and LR scan of the LRNC area, the FC thickness, the calcified plaque area and the loose matrix plaque areas of the HR scans. Likewise, no significant differences were found between the HR and LR rescan of the LRNC area, the FC thickness, the calcified plaque area and the loose matrix plaque areas of the LR scans. Fig 3 shows the Bland-Altman plots for the LRNC area, the FC thickness, the calcified plaque area and loose matrix area measurements between the scan and rescan for the open and closed segmentation method of the HR and LR scans. The ICC of the HR LRNC area with the closed segmentation was 0.58 (0.0–0.81), indicating a fair to good scan-rescan reproducibility. All other ICCs of the HR and LR scans and rescans for the open and closed segmentation method were greater than 0.75 (Tables 4–6). No significant differences using a Levene’s test were found in the measurement variability for the LRNC area, the FC thickness, the calcified plaque area and the loose matrix plaque areas between the HR scan and rescan or the LR scan and rescan. Likewise, using a Levene’s test no significant differences in the measurement variability for the LRNC area, the FC thickness, the calcified plaque area and the loose matrix plaque areas between the open and closed segmentation method were found.

### Within-reader and between-reader reproducibility

The ICC values for the within-reader reproducibility and the between-reader reproducibility of the LRNC area, the calcified plaque area and loose matrix area measurements are given in Table 8. No significant differences were found between the HR and LR within-reader and between-reader reproducibility of the carotid MRI dimension measurements.

**Table 8:** Within-reader and between-reader reproducibility for the HR and LR carotid arterial wall component measurements. Data are presented as mean with (95% CI). Last column contains p-value for Levene’s test between HR and LR measurements. HR = high resolution; LR = low resolution.

	HR	LR	p
Between-reader reproducibility			
Lipid	0.66 (0.41–0.80)	0.40 (0.0–0.66)	0.15
Calcium	0.92 (0.87–0.96)	0.83 (0.71–0.91)	0.46
Loose matrix	0.63 (0.37–0.79)	0.71 (0.48–0.83)	0.07
Within-reader reproducibility			
Lipid	0.63 (0.36–0.78)	0.85 (0.74–0.92)	0.33
Calcium	0.91 (0.84–0.95)	0.97 (0.95–0.99)	0.15
Loose matrix	0.77 (0.60–0.76)	0.89 (0.81–0.94)	0.19

## Discussion

This study shows that increasing the spatial resolution of carotid MRI above the commonly used 0.50 × 0.50 mm^2^ in-plane resolution does not improve scan-rescan reproducibility of atherosclerotic plaque components. These findings are in contrast to a recent paper demonstrating an improved scan-rescan reproducibility for carotid plaque dimension measurements using 3T carotid MRI.[25] Furthermore, we could not demonstrate any difference in scan-rescan reproducibility between the closed and open segmentation method. No differences between the within-reader and between-reader reproducibility between the HR and the LR carotid plaque component measurements were found. Measurements of calcified and loose matrix plaque area show an excellent between-reader and within-reader reproducibility. The exclusion rates of our study are lower[14] or similar[13] compared to other studies.

Comparing our scan-rescan reproducibility data with previous studies is hindered by the heterogeneous setup of the various studies previously performed. Saam et al. investigated the reproducibility of interscan plaque composition analyzing the scans simultaneously with an in-plane resolution of 0.50 × 0.50 mm^2^ in 20 patients at 1.5 T.[12] Baseline carotid dimension measurements (normalized wall index 0.6 ± 0.1), were similar to our population characteristics (normalized wall index 0.7 ± 0.1), making a comparison possible with the open LR scan-rescan reproducibility data. ICCs for calcification measurements (ICC 0.95, CI0.9–1.0) and the LRNC measurements (ICC 0.99, CI 0.98–1.00) were within the same range as our LR open segmentation ICCs for calcified plaque (ICC 0.99, CI 0.99–1.00) and the LRNC plaque area (ICC 0.98, CI0.90–0.99). The more recent study of Li et al. conducted repetitive scans within 14 days in 20 patients at 3T MRI with an in-plane resolution of 0.55 × 0.55 mm^2.^ Scans were analyzed in a manner that resembles our open segmentation method, since the calcified and LRNC plaque composition analyses were performed using only those arteries that exhibited the plaque composition in at least one time point. Different baseline characteristics (normalized wall index 0.33± 0.1; mean wall thickness 0.86 ± 0.19) indicate this study was performed in less diseased patients, which is further corroborated by the wider selecting criteria, including patients with a carotid stenosis > 15%, hindering a direct comparison. Although the scan-rescan reproducibility of FC status assessment has been published previously, important differences in study design make a direct comparison impossible. In the study of Kwee et al. FC status was assessed and therefore a population was selected with a recent cerebral vascular event[26]. To the best of our knowledge no FR thickness measurement reproducibility data have been published previously in a population comparable to our patients. In our population we could not determine the scan-rescan reproducibility of intraplaque hemorrhage due to the low prevalence of intraplaque hemorrhages in our population which consisted of patients with stable cardiovascular disease in contrast to previous data which was predominantly obtained from patients after a stroke[10,13]. Alternatively, the absence of T1w-GRE sequence in our protocol may also have decreased the number of cases with intraplaque hemorrhages[27].

The lack of improved plaque component scan-rescan reproducibility at higher resolution in our data indicate that the decrease in CNR and SNR, especially noticeable on the T2w images, outweighed the advantages of the higher spatial resolution. Furthermore, the high resolution scan was considerably longer at 1.5 times more scantime. Future developments in MR coil design, fast imaging and reconstruction methods or the use of 7T MRI may compensate for the loss in SNR at higher resolutions [28], enabling the use of higher resolution for carotid MRI.

No significant improvement between the ICCs of the open segmentation method compared to the ICCs of the closed method could be demonstrated. However, lower confidence intervals for the ICCs of the open segmentation method indicate that a beneficial effect may be present in a larger population suggesting that the open segmentation method is the preferable choice for carotid MRI component analyses. The larger CIs in the closed read might be explained, at least partially, by the scan-rescan plaque component identification mismatch (component identified in the scan or rescan, but not in the rescan or scan). Although mismatches occurred less frequent with the open segmentation method, repeated images with mismatches were included in the final analyses, in contrast to previously published studies.[6,12] Taken together, our data suggest that future clinical intervention trials using carotid plaque imaging as surrogate endpoint can reduce the impact of these issues by reviewing scans with an open segmentation method fully blinded for intervention and time-point.

Literature on accuracy for carotid artery plaque composition using different resolutions with carotid MRI is lacking or scarce. Keenan et al. recently described a 7% larger area in low resolution (0.43 × 0.43 mm^2^) measurements compared to high resolution measurements (0.195 × 0.190 mm^2^) in an ex vivo study validating vessel wall size with histology.[29] Similar findings of overestimated dimension measurements were also demonstrated by comparing vessel wall MR imaging (0.78 × 0.49 mm^2^) with high resolution intravascular ultrasonography.[30] Using a low and high resolution scanning protocol, without any changes in coils, scan sequence parameters, patient population and other factors potentially affecting scan comparisons we find that 88.9% of all mean HR scans (scan, rescan and second observer) had a lower plaque component surface area compared to the mean LR scans. More specifically, the significant lower surface area for plaque loose matrix composition with the HR scanning protocol suggests that increasing the spatial in-plane resolution improves plaque loose matrix composition measurement accuracy, which is probably due to a reduced partial volume effect at the increased resolution.

### Limitations

A limitation of the current study is that 2D sequences are compared whereas nowadays 3D isotropic sequences are used more often. We anticipate that similar results would have been obtained if 3D sequences with varying voxel sizes would have been compared[31].

## Conclusion

Our data indicate that the scan-rescan reproducibility of carotid artery plaque composition with an open segmentation method is excellent. Increasing the spatial resolution at the expense of the contrast-to-noise ratio above the routinely used 0.50 × 0.50 mm^2^ does not improve measurement reproducibility for carotid plaque component measurements contrary to earlier findings for plaque dimension measurements [17]. We believe these differences can be explained by the different size of a vessel wall compared to the size of a component within the vessel wall, which is significantly smaller making it much more difficult to obtain a good scan-rescan reproducibility. Second, we believe that since component analyses are dependent on a good quality of multiple different weighted images the results are much more sensitive to image artefacts than dimension measurements where the vessel wall dimension can be more easily be acquired on different sequences (e.g. T1w, or PDw sequences).

Clinical intervention trials using plaque component areas as surrogate endpoint can decrease their subject number by using an optimal scan-rescan reproducibility for plaque component measurement. Our data indicate that LR measurements on a 3T MRI scanner equipped with a dedicated carotid coil combined with an open segmentation method are the optimal choice in such a setting.
